# Radiotherapy dose distribution prediction for breast cancer using deformable image registration

**DOI:** 10.1186/s12938-020-00783-2

**Published:** 2020-05-29

**Authors:** Xue Bai, Binbing Wang, Shengye Wang, Zhangwen Wu, Chengjun Gou, Qing Hou

**Affiliations:** 1 Key Laboratory of Radiation Physics and Technology, Ministry of Education, Institute of Nuclear Science and Technology, Sichuan University, Chengdu, 610064 China; 2 Institute of Cancer and Basic Medicine (ICBM), Chinese Academy of Sciences, Hangzhou, 310022 China; 3 Department of Radiation Physics, Cancer Hospital of the University of Chinese Academy of Sciences, Hangzhou, 310022 China; 4 Department of Radiation Physics, Zhejiang Cancer Hospital, Hangzhou, 310022 China

**Keywords:** Radiotherapy, Deformable image registration, Dose prediction, Breast cancer

## Abstract

**Background:**

Radiotherapy treatment planning dose prediction can be used to ensure plan quality and guide automatic plan. One of the dose prediction methods is incorporating historical treatment planning data into algorithms to estimate the dose–volume histogram (DVH) of organ for new patients. Although DVH is used extensively in treatment plan quality and radiotherapy prognosis evaluation, three-dimensional dose distribution can describe the radiation effects more explicitly. The purpose of this retrospective study was to predict the dose distribution of breast cancer radiotherapy by means of deformable registration into atlas images with historical treatment planning data that were considered to achieve expert level. The atlas cohort comprised 20 patients with left-sided breast cancer, previously treated by volumetric-modulated arc radiotherapy. The registration-based prediction technique was applied to 20 patients outside the atlas cohort. This study evaluated and compared three different approaches: registration to the most similar image from a dataset of individual atlas images (SIM), registration to all images from a database of individual atlas images with the average method (WEI_A), and the weighted method (WEI_F). The dose prediction performance of each strategy was quantified using nine metrics, including the region of interest dose error, 80% and 100% prescription area dice similarity coefficients (DSCs), and γ metrics. A Friedman test and a nonparametric exact Wilcoxon signed rank test were performed to compare the differences among groups. The clinical doses of all cases served as the gold standard.

**Results:**

The WEI_F method could achieve superior dose prediction results to those of WEI_A. WEI_F outperformed SIM in the organ-at-risk mean absolute difference (MAD). When using the WEI_F method, the MAD values for the ipsilateral lung, heart, and whole lung were 197.9 ± 42.9, 166 ± 55.1, 122.3 ± 25.5, and 55.3 ± 42.2 cGy, respectively. Moreover, SIM exhibited superior prediction in the DSC and γ metrics. When using the SIM method, the means of the 80% and 100% prescription area DSCs, 33γ metric, and 55γ metric were 0.85 ± 0.05, 0.84 ± 0.05, 0.64 ± 0.13, and 0.84 ± 0.10, respectively. The plan target volume and spinal cord MAD when using SIM and WEI were 235.6 ± 158.4 cGy versus 227.4 ± 144.0 cGy ($p > 0.05$) and 61.4 ± 44.9 cGy versus 55.3 ± 42.2 cGy ($p > 0.05$), respectively.

**Conclusions:**

A predicted dose distribution that approximated the clinical plan could be generated using the methods presented in this study.

## Background

Breast cancer is the most common malignancy in women, ranking first in female morbidity and mortality [1]. The main treatment method of early-stage breast cancer is multidisciplinary comprehensive treatment consisting of surgery, chemotherapy and radiotherapy [2, 3]. Regarded as a standard treatment method for early-stage breast cancer, total breast irradiation after breast-conserving surgery can effectively improve local control rate and long-term survival rate [4].

Although two-dimensional tangent irradiation and three-dimensional conformal radiotherapy have been confirmed appropriate therapy for breast cancer, new techniques are developed to achieve better target coverage and normal tissues spare. New irradiation methods, intensity-modulated radiotherapy (IMRT) and volumetric-modulated arc therapy (VMAT) have been introduced. Compared to former techniques, the IMRT and VMAT can deliver a more uniform and conformity dose distribution inside the target and a superior protection of organs of interest (OARs) [5, 6]. The quality of the treatment plan depends on the algorithms of the treatment planning system (TPS), the delivery techniques, and in particular, the experience and skill of the planner [7].

Traditionally, the criteria of the dose limitation and target coverage of normal tissues have followed the Radiation Therapy Oncology Group guidelines, which use a set of uniform standards for patients with same-sited tumors. However, owing to the large anatomy variations among individuals, while uniform standards may be suitable for several patients, they are always either easily exceeded or unachievable for others. Planners generally require sufficient trail-and-error time to avoid the risk of degraded plan quality and to generate an optimal plan. Moreover, peer review is recommended to ensure the consistency of the plan quality among different planners and institutions [8]. Although such procedures can effectively improve the quality of radiotherapy, with the development of new technology, such as remote radiotherapy, a rapid and an automatic individualized evaluation tool is urgently required.

To improve the current situation of patient-specific achievable criteria deficiencies, several studies have focused on incorporating historical treatment planning data that were considered to have achieved expert level into algorithms to estimate the organ dose for new patients [9–15]. These researches explain that predicted dose values could be used to estimate the plan quality and guide-automated treatment planning. However, most of those studies were devoted to predicting the dose–volume histogram (DVH), which is used extensively in treatment plan quality and radiotherapy prognosis evaluation, but remains limited. At present, oncologists and physicists are attempting to describe the radiation effects more explicitly, using models related to three-dimensional (3D) dose distribution geometry, such as normal tissue complication probability [16] and 3D cluster formation [17].

In recent years, several studies have been concerned with the prediction of dose distribution from patient computed tomography (CT) images and regions of interest (ROIs) [18–24]. Shiraishi and Moore used several voxel parameters that indicate the volume and location of ROIs to train artificial neural networks for prostate and stereotactic radiosurgery/radiotherapy cases, and predicted dose distributions at the area nearby plan target volume (PTV) boundary [18]. With the fully convolutional network, contoured voxel matrix and historical dose matrix were input into the network to train the prediction models, the features were selected and extracted automatically by networks. The deep learning-based methods are commonly used to predict 3D dose distribution for prostate, head and lung cancer radiotherapy [19, 20, 22].

With a well-trained model, deep learning-based methods could predict dose prediction in seconds, but the training process takes a lot of time and needs amount of data. Moreover, the interpretation deficiency of end-to-end deep learning network obstructs its application in clinic. Compared with deep learning-based methods, atlas-based methods could be implemented with less prepared cases, which was more flexible for institutions to build their own atlas dataset for each treatment site. The atlas-based approach has been successfully used in automatic medical image segmentation [25]. McIntosh and Purdie used an atlas-based method to transfer dose distribution from historical plans to a new patient for three sites containing whole breast IMRT, but such methods have not been extensively investigated [19]. Yoganathan and Zhang further elaborate the 3D dose prediction model based on atlas and deformable image registration (DIR) for VMAT breast and prostate cancer patients [21]. They arbitrarily chosen a reference patient from dataset and registered other patients’ CT to the chosen one. A test patient was registered to the reference one and the deform vector filed was used to determine the similarity weight between new patient and atlas patients. Thereafter, the predicted dose was obtained using weighted summation of deformed atlas doses.

The purpose of this work was to further investigate the atlas-based dose prediction method for breast cancer. We used three variation atlas-selection models to predict the 3D dose distribution for breast patients, and the predicted results were compared with the clinical plans. The 3D moment invariants (3DMI), which are often used in visual pattern recognition, were first used to select atlas images in this study.

## Results

### Feature selection

There were a total of 190 CT volume image pairs for all 20 atlas cases. In each pair, one image was defined as the atlas image in turn, and the other was defined as the new image, so that 380 groups could be used for statistical analysis. The predicted dose distribution for the new image was calculated from the atlas image and compared to the clinical plan dose. The results of the Spearman’s rank correlation between the geometric features and dosimetric errors are listed in Table 1.

**Table 1:** Spearman’s rank correlation test

	PTV	Ipsilateral lung	Heart	Lung	Spinal cord
Image similarity metric					
$\rho$	0.378*	0.307*	0.191*	0.431*	0.395*
*p*	0.000	0.000	0.000	0.000	0.000
PTV DSC					
$\rho$	0.365*	0.333*	0.275*	0.408*	0.392*
*p*	0.000	0.000	0.000	0.000	0.000
Ipsilateral lung OVH MSD					
$\rho$	0.197*	0.236*	0.155*	0.215*	0.005
*p*	0.000	0.000	0.000	0.000	0.914
Heart OVH MSD					
$\rho$	0.142*	0.160*	0.102*	0.172*	0.234*
*p*	0.005	0.001	0.041	0.001	0.000
PTV length difference					
$\rho$	0.293*	0.338*	0.355*	0.249*	0.209*
*p*	0.000	0.000	0.000	0.000	0.000
$${\text{P}}_{\text{ovz}}$$					
$\rho$	0.191*	0.428*	0.395*	0.369*	0.356*
*p*	0.000	0.000	0.000	0.000	0.000
$$J_{{1\_{\text{diff}}}}$$					
$\rho$	0.135*	0.280*	0.131*	0.343*	0.269*
*p*	0.007	0.000	0.009	0.000	0.000
$$J_{{ 2 {\text{\_diff}}}}$$					
$\rho$	0.111*	0.222*	0.095	0.316*	0.239*
*p*	0.026	0.000	0.057	0.000	0.000
$$J_{{ 3 {\text{\_diff}}}}$$					
$\rho$	0.088	0.065	0.022	0.180*	0.289*
*p*	0.080	0.196	0.658	0.000	0.000
$$B_{{ 3 {\text{\_diff}}}}$$					
$\rho$	0.137*	0.087	0.054	0.240*	0.314*
*p*	0.006	0.081	0.282	0.000	0.000
$$B_{{ 4 {\text{\_diff}}}}$$					
$\rho$	0.192*	0.221*	0.165*	0.351*	0.350*
*p*	0.000	0.000	0.001	0.000	0.000

	0.8 DSC	1.0 DSC	33 $\gamma$	55 $\gamma$
Image similarity metric				
$\rho$	− 0.468*	− 0.484*	− 0.282*	− 0.392*
*p*	0.000	0.000	0.000	0.000
PTV DSC				
$\rho$	− 0.663*	− 0.655*	− 0.271*	− 0.321*
*p*	0.000	0.000	0.000	0.000
Ipsilateral lung OVH MSD				
$\rho$	− 0.235*	− 0.281*	− 0.249*	− 0.242*
*p*	0.000	0.000	0.000	0.000
Heart OVH MSD				
$\rho$	− 0.195*	− 0168*	− 0.065	− 0.087
*p*	0.000	0.001	0.195	0.084
PTV length difference				
$\rho$	− 0.405*	− 0.327*	− 0.343*	− 0.319*
*p*	0.000	0.000	0.000	0.000
$${\text{P}}_{\text{ovz}}$$				
$\rho$	− 0.455*	− 0.368*	− 0.282*	− 0.271*
*p*	0.000	0.000	0.000	0.000
$$J_{{ 1 {\text{\_diff}}}}$$				
$\rho$	− 0.231*	− 0.245*	− 0.174*	− 0.203*
*p*	0.000	0.000	0.000	0.000
$$J_{{ 2 {\text{\_diff}}}}$$				
$\rho$	− 0.214*	− 0.231*	− 0.152*	− 0.182*
*p*	0.000	0.000	0.002	0.000
$$J_{{ 3 {\text{\_diff}}}}$$				
$\rho$	− 0.193*	− 0.202*	− 0.051	− 0.089
*p*	0.000	0.000	0.314	0.075
$$B_{{ 3 {\text{\_diff}}}}$$				
$\rho$	− 0.223*	− 0.201*	− 0.066	− 0.132*
*p*	0.000	0.000	0.186	0.008
$$B_{{ 4 {\text{\_diff}}}}$$				
$\rho$	− 0.348*	− 0.310*	− 0.200*	− 0.243*
*p*	0.000	0.000	0.000	0.000

* Represents significant correlation

As all 11 features were significantly correlated with most of the dose prediction result measures, none of these was excluded for further analysis. Thereafter, the features were tested using the KMO metric and Bartlett’s sphericity test, which yielded results of 0.734 and *p* = 0.000, respectively, demonstrating that these 11 features exhibited correlation, and factor analysis could be used to decrease the feature number. The number of variables used to characterize the patient geometry was reduced from 11 to 4 by applying factor analysis. Using the Kaiser eigenvalues criterion, four factors were extracted, with eigenvalues of 4.124, 1.843, 1.422, and 1.022. These factors collectively explained 76.47% of the variance in all 11 original features. The results of the feature factor analysis are listed in Table 2.

**Table 2:** Rotated component matrix of factor analysis

	Factor			
	$F_{1}$	$F_{2}$	$F_{3}$	$F_{4}$
Image similarity metric	0.411	− 0.040	0.493	0.323
PTV DSC	0.245	− 0094	0.578	0.308
Ipsilateral lung OVH MSD	0.076	0.878	0.145	− 0.138
Heart OVH MSD	0.012	0.137	0.030	0.891
PTV length difference	− 0.101	0.074	0.816	− 0.082
${\text{P}}_{\text{ovz}}$	0.161	0.098	0.792	− 0.083
$J_{{ 1 {\text{\_diff}}}}$	0.963	0.099	0.115	0.016
$J_{{ 2 {\text{\_diff}}}}$	0.955	0.113	0.067	0.035
$J_{{ 3 {\text{\_diff}}}}$	0.495	0.693	− 0.029	0.346
$B_{{ 3 {\text{\_diff}}}}$	0.177	0.764	− 0.077	0.489
$B_{{ 4 {\text{\_diff}}}}$	0.834	0.273	0.173	0.085

Considering that the features with component values larger than 0.7 were the main components for each factor, $F_{1}$ was mainly composed of $J_{{1\_{\text{diff}}}}$, $J_{{2\_{\text{diff}}}}$, and $B_{{4\_{\text{diff}}}}$. As the 3DMIs of the three features represented the PTV shape, $F_{1}$ could be interpreted as a PTV shape factor. $F_{2}$ was mainly composed of the ipsilateral lung OVH MSD and $B_{{3\_{\text{diff}}}}$, and the factor represented the spatial relationship between the PTV and ipsilateral lung. As the main components of $F_{3}$ were the PTV length difference and ${\text{P}}_{\text{ovz}}$, this factor could be explained as a PTV consistency factor on the z-axis. The main components of $F_{4}$ were the image similarity metric and PTV DSC, in that this factor could be explained as a global consistency factor. The four factors were then combined into a comprehensive score using Eq. (9).

### Dose prediction validation

Three dose prediction algorithms were evaluated. The atlas-selection method was examined using similarity selection (SIM) and all images with a weighted combination (WEI). For the similarity selection, the comprehensive score $F$ was used as the selection gauge. For all images with weighted combinations, the weight was examined using the average weight (WEI_A) and metric weight by means of Eq. (12) (WEI_F). The quantitative MAD results are presented in Table 3.

**Table 3:** Quantitative MAD results with standard deviation σ of three dose prediction methods

Method	PTV (cGy)	Ipsilateral lung (cGy)	Heart (cGy)	Lung (cGy)	Spinal cord (cGy)
SIM					
Mean	235.6	250.7	202.8	152.9	61.4
*σ*	158.4	68.2	64.4	47.7	44.9
WEI_A					
Mean	267.5	213.0	174.1	130.9	57.1
*σ*	176.4	50.8	55.7	26.2	39.9
WEI_F					
Mean	227.4	197.9	166.0	122.3	55.3
*σ*	144.0	42.9	55.1	25.5	42.2
*p*	0.004*	0.002*	0.002*	0.002*	0.116
SIM–WEI_ A					
*p*	0.627	0.015*	0.019*	0.073	–
SIM–WEI_F					
*p*	0.232	0.002*	0.006*	0.012*	–
WEI_ A–WEI_F					
*p*	0.000*	0.001*	0.001*	0.001*	–

Method	0.8 DSC	1.0 DSC	33 $\gamma$	55 $\gamma$
SIM				
Mean	0.85	0.84	0.64	0.84
*σ*	0.05	0.05	0.13	0.10
WEI_A				
Mean	0.86	0.82	0.55	0.75
*σ*	0.05	0.08	0.14	0.12
WEI_F				
Mean	0.87	0.84	0.58	0.78
*σ*	0.05	0.07	0.15	0.13
*p*	0.015*	0.002*	0.000*	0.000*
SIM–WEI_A				
*p*	0.135	0.156	0.002*	0.001*
SIM–WEI_F				
*p*	0.017*	0.654	0.015*	0.015*
WEI_A–WEI_F				
*p*	0.002*	0.000*	0.000*	0.000*

* Represented significant correlation

All of the dose error metrics exhibited significant differences among the groups of prediction methods, except for the MAD of the spinal cord. The results of the post hoc analysis of the Wilcoxon signed rank test for the other dose error metrics are presented in Table 3. The results demonstrated that using the WEI_F method could achieve superior dose prediction results to those of WEI_A. Compared to WEI_F, the SIM method exhibited inferior performance in the ipsilateral lung, heart, and lung MADs, but superior performance in the 0.8 DSC, 33γ, and 55γ.

## Discussion

Atlas selection and image registration have been successfully applied in automatic image segmentation, providing highly accurate results [26, 27]. The new segmentation can be transformed from atlas segmentation by means of the transformation generated from the registration between the new and atlas images. This method is dependent on the strong correlation between medical images and organ segmentation. In radiotherapy, two patients with identical medical images should intuitively be treated with the same plan, and the dose distribution for a special patient depends on the relationship of the target–normal tissue position. However, unlike in image segmentation, many influencing factors exist from the image to planning the dose distribution; for example, the delivery technique, beam orientation, dose computation algorithm, optimized algorithm, and skill of the planner. To limit these influencing factors, patients enrolled in this study were treated with the same delivery technique (VMAT), dose computation algorithm, and optimized algorithm in identical TPSs. Under these circumstances, the most uncertain factors affecting the dose distribution were the planner experience and skill, containing the beam angles, and optimizing the parameter selection. Although the correlation between the medical image and dose distribution was more complex and less direct, the feasibility of using DIR to generate patient-specific dose distributions for radiotherapy was validated in this study.

In this study, three atlas-selection methods were tested for 3D dose prediction. A total of 11 objective similarity metrics for different patients constituted a comprehensive score $F$, which was used in WEI_F to reduce the probabilistic atlas to a deterministic atlas. The results of WEI_F were superior to those of WEI_A, demonstrating that the $F$ calculation method used in this study was reasonable, and the atlas image that is more similar to the new image should receive a larger weight. WEI_F outperformed SIM in the OAR MADs, with the exception of the spinal cord, while SIM could provide superior prediction in the DSC and γ metrics. The OAR MAD evaluated the area outside the PTV, while the DSC and γ metrics focused more on the high-dose area, which is near and inside the PTV boundary. As the SIM method only used the information of the most similar single atlas images, and WEI_F considered all of the cases in the dataset, the results implied that the dose distribution in the area of the dose higher than 80% of the prescription dose was more strongly related to the patient-specific geometry than the lower dose area. This is reasonable because, in a VMAT plan, dose drops rapidly in the region near the PTV margin, which causes the dose distribution to vary substantially among individual patients. Moreover, our dose prediction methods registered images with PTV constraints, causing the algorithm to focus more on the high-dose area. The dose distribution usually exhibits a steep gradient in the area near the PTV boundary, and becomes flat with the voxels far from the PTV. The MAD of the spinal cord indicated no significant difference among the three methods. Because the spinal cord has a greater distance to the PTV than the other organs investigated in this study, the dose distribution in the spinal cord is more flat and less individual for different patients, and the atlas-selection methods have little impact on the spinal cord dose prediction.

**Fig. 1:**
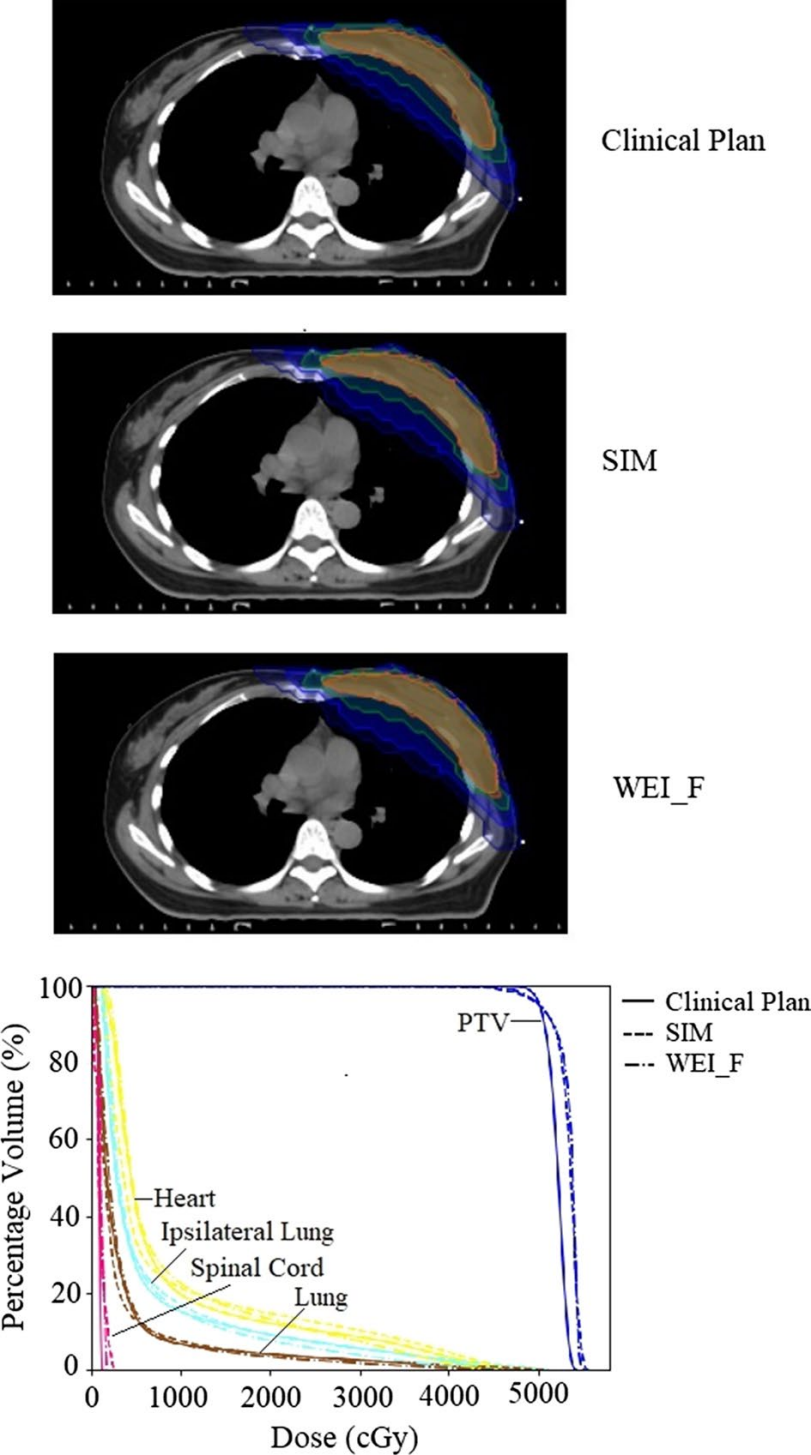
Comparison of clinical plan and predicted dose distributions, with corresponding DVHs

Figure 1 presents an example of the comparison of the clinical plan and predicted dose distributions with the corresponding DVH. As WEI_A exhibited inferior performance to WEI_F for all of the evaluation metrics, the WEI_A results were excluded from further discussion. In this case, the SIM and WEI_F methods could provide a dose distribution that approximated that of the clinical plan. The number and quality of cases in atlas database were the crucial factors for predicted results. Constructing an atlas database containing variation images would be helpful for obtaining improved prediction results. To enhance the prediction model capability and obtain more reliable validation results, our future work will focus on collecting more patient cases and investigating the optimal number for the atlas database. Expanding the database could increase the accuracy of the framework with the increased patient diversity.

The general findings of this study are consistent with previous literatures. The summary of breast cancer radiotherapy dose prediction results is shown in Table 4. Although the different patient datasets and treatment protocols that exist prevented direct comparisons with our study, we demonstrated the feasibility of accurately predicting the dose distributions for breast cancer treatment with purposed methods.

**Table 4:** Summary of breast cancer radiotherapy dose prediction results

	McIntosh and Purdie [19]	Yoganathan and Zhang [21]	Our’s	
Methods	Contextual atlas regression forest	–	SIM	WEI_F
Training/atlas cases	144	19	20	20
55 $\gamma$	0.79 ± 0.08	–	*0.84 ± 0.10*	0.78 ± 0.13
0.8 DSC	0.86 ± 0.05	–	0.85 ± 0.05	*0.87 ± 0.05*
MAD over entire CT volume (cGy)	–	900 ± 110	*48.26 ± 226.90*	*42.45 ± 181.62*

The better results were printed in italics. Notice that the results were based on different patient datasets and treatment protocols

To the best of the authors’ knowledge, this is the first study on DIR-based dose prediction using 3DMI. Compared with structural similarity index used by Yoganathan and Zhang [21], which was calculated over a 2D slice and was averaged to obtain a 3D metric, the 3DMI could quantify the shape of 3D structure directly and accurately. Beside of the multi-atlas method used in previous studies, our study evaluates the performance of single-atlas method in dose prediction. The results demonstrated the single-atlas performed superior in high-dose area than multi-atlas method.

The 40 involved plans were made by different dosimetrists. Since all plans were satisfying the institutional critical and reviewed by two senior dosimetrists and one oncologist, the variation between planers was supposed few and ignored in the prediction result evaluation. However, for a specific case, subjective fluctuations remained owing to the preferences of each planner. Several different plans could be all considered as achieving expert level. These plans were a series of Pareto optimized plans, which could be composed into the Pareto front [28–30]. Therefore, in certain cases, although the predicted dose distribution exhibited errors compared to the clinical plan, it could be acceptable in clinical applications. The feasibility of generating a series of dose distributions on the Pareto front with DIR will be tested and validated in our future work.

There are several limitations to this study. First, the methods were applied on left-breast cancer only and the validation dataset was limited. The next phase of our study will involve more validation cases, other sites and multiform delivery techniques. Second, although the predicted results were close to the clinical accepted plans, it could not be guaranteed as the best achievable. Further validations were needed to determine whether the predicted results could be used for plan quality control. Third, the present study has not tested whether the predicted dose distribution could be transformed to treatment parameters and be used to generate clinical plan. This work is a foundation for further study, and related automatic plan research would be conducted in our future work.

## Conclusions

This study investigates the atlas-based dose prediction method for breast cancer treated using VMAT. The 3DMI were first used to select atlas images for dose prediction in proposed method. The method has presented an achievable dose distribution prediction framework based on DIR. With different atlas-selection approaches, SIM exhibited superior prediction in target region while WEI_F outperformed other methods in OARs. Constructing an atlas database containing variation images is helpful for obtaining improved prediction results. The achievable dose distributions that were obtained from proposed prediction framework provided an effective foundation for treatment plan quality assurance, and for guiding automatic planning to reduce the planning time.

## Methods

The purpose of this retrospective study was to predict the achievable radiotherapy dose distribution for early-stage left-sided breast cancer patients. The proposed dose prediction framework included three major steps: database building, atlas selection, and deformable registration. In the first step, a dataset was constructed with historical cases containing patient CT images and expert plans. Thereafter, an initial alignment of the new images and all images in the dataset was achieved using a rigid registration method. Finally, the atlas image was selected from the dataset and registered to a new image, and the registration displacement could be used to generate the predicted dose distribution for the new patient.

### Database

A total of 40 randomly selected patients with left-sided breast cancer (stage T1M0N0), previously treated using VMAT at Zhejiang Cancer Hospital (Zhejiang, China) from 2016 to 2018, were enrolled in this study. Clinical treatment plans were generated for all patients using the RayStation (RaySearch Laboratories, Stockholm, Sweden) TPS, with 6 MV X-rays on a Trilogy Linear Accelerator (Varian Medical Systems, Palo Alto, USA). The prescription dose was 5000 cGy (200 cGy/fraction); the oncologist delineated the clinical target volume (CTV), PTV, heart, left lung, whole lung, and spinal cord. The CTV encompassed the visible breast tissue and tumor bed. The PTV was constructed by adding a 10-mm margin to the CTV for all plans. All PTVs were clipped 5 mm from the skin surface. The VMAT plans were delivered using double arcs with a gantry spacing of 4° between control points, and the beam range was adapted to the patient-specific situation. The basic characteristics of patients, contours, and plans are listed in Table 5. All plans were optimized further using a trial-and-error process to achieve optimal sparing of normal tissues. Two experienced dosimetrists and one senior oncologist at Zhejiang Cancer Hospital reviewed the plans. Normal tissues in all plans conformed the as low as achievable principle. In this study, 20 cases were chosen randomly and comprised the library of previously planned cases (atlases). Other 20 cases were used as a test set for validation. The data involved in this study were categorized into three 3D matrices: CT image, ROI labels, and dose distribution. It was achieved using functions in the SimpleITK of python [31, 32].

**Table 5:** Basic characteristics of patients, contours, and plans

	Average	Range
Age	57	49–62
Body mass index	21.3	19.7–24.7
PTV volume (cm^3^)	628	325–1185
Heart volume (cm^3^)	558	383–714
Ipsilateral lung volume (cm^3^)	995	607–1539
Whole lung volume (cm^3^)	2224	1373–3299
Beam start angle (°)	300	290–310
Beam stop angle (°)	133	125–145

### Image registration

In a given image registration frame, a fixed image was defined as the object that was assumed to be static, while a floating image was defined as the object that would be transformed to be superimposed onto the target image. The matrices of the fixed and floating images were denoted by $$\overset{\lower0.5em\hbox{$\smash{\scriptscriptstyle\rightharpoonup}$}} {I}_{\text{fixed}}$$ and $$\overset{\lower0.5em\hbox{$\smash{\scriptscriptstyle\rightharpoonup}$}} {I}_{\text{floating}}$$ in a particular registration. The registration algorithm used in this study consisted of three major steps. First, rigid registration was used to bring two images into global correspondence. Thereafter, based on the linear transformation result of the rigid registration, the reference image was deformed with nonlinear transformation to achieve local correspondence to the target image. Finally, as the PTV received the greatest optimization weight during the treatment plan design, the floating image would be further deformed using PTV as a task-specific constraint.

In the first step, initial alignment of the images was achieved using a similarity 3D transform rigid registration method, whereby a rotation (with three angles), a translation (with three vectors), and isotropic scaling (with one factor) were applied to the space. The mean squared deviation (MSD) criterion based on image gray value was used as an optimization metric [33]. MSD is zero in case of the floating image exactly coinciding with the fixed image. The rigid transformation was denoted by $$f_{R}$$, and $$f_{R} \left( {\overset{\lower0.5em\hbox{$\smash{\scriptscriptstyle\rightharpoonup}$}} {I}_{\text{floating}} } \right)$$ was used as the initial condition for the deformable registration in the following step.

Based on the rigid registration, local image deformation was achieved using the Demons deformable registration, which is a widely used image registration method in radiotherapy [34]. The deformable transformation was denoted by $$f_{D}$$. In addition to the image intensity, the PTV region was considered as a constraint area by means of further optimization. The delineated PTVs of the floating and fixed images were extracted as region masks. In the mask images, voxels belonging to PTV have value 1 while other voxels have value 0. A further transformation $$f_{P}$$ was generated through registration of the mask images. Hence, $$f_{D} \left( {f_{R} \left( {\overset{\lower0.5em\hbox{$\smash{\scriptscriptstyle\rightharpoonup}$}} {I}_{\text{floating}} } \right)} \right)$$ represented the registration result without the PTV constraint, while the result with the PTV constraint could be obtained by $$f_{P} \left( {f_{D} \left( {f_{R} \left( {\overset{\lower0.5em\hbox{$\smash{\scriptscriptstyle\rightharpoonup}$}} {I}_{\text{floating}} } \right)} \right)} \right)$$. All the above methods were implemented using the library from the SimpleITK system [31, 32].

### Patient similarity metrics

#### Geometry features

Similarity metrics between two patients were required to select the atlas image. In total, 11 features were extracted to characterize the different scales of the patient geometry, which are described in the following text. (1) The MSD was used as the image similarity metric, which described the global difference between two images. (2) The dice similarity coefficient (DSC) of two PTVs was used to describe the PTV similarity. The DSC was defined as

1 $${\text{DSC}} = \frac{{2\left( {V_{PTV1} \cap V_{PTV2} } \right)}}{{V_{PTV1} + V_{PTV2} }},$$

where $V_{PTV1}$ is the PTV area in the atlas image and $V_{PTV2}$ is the PTV area in the new image. The DSC was calculated after rigid alignment between two images; ${\text{DSC}} = 1$ when PTV1 and PTV2 coincided with one another, and ${\text{DSC}} = 0$ when the two areas did not intersect. (3) The overlap volume histogram (OVH) is a general, sophisticated, and robust shape relationship descriptor associated with an OAR, measuring its proximity to a target [9]. The OVH value represents the percentage of the OAR volume that overlaps with a uniformly expended target. As the dose constraints of the ipsilateral lung and heart are the most important factors that affect the results of left-breast tangent VMAT plans, the MSD of the left lung–PTV OVH (MSD_OVH_) and (4) MSD_OVH_ of the heart–PTV were considered as features to describe the geometric relationship of the OARs and PTVs. The MSD_OVH_ was calculated using

2 $${\text{MSD}}_{\text{OVH}} = \frac{1}{99}\mathop \sum \limits_{i = 1}^{99} \left( {\left| {O1_{i\% } - O2_{i\% } } \right|} \right),$$

where $O1_{i\% }$ and $O2_{i\% }$ are the overlap values at i % volume of the atlas case and the new cases.

**Fig. 2:**
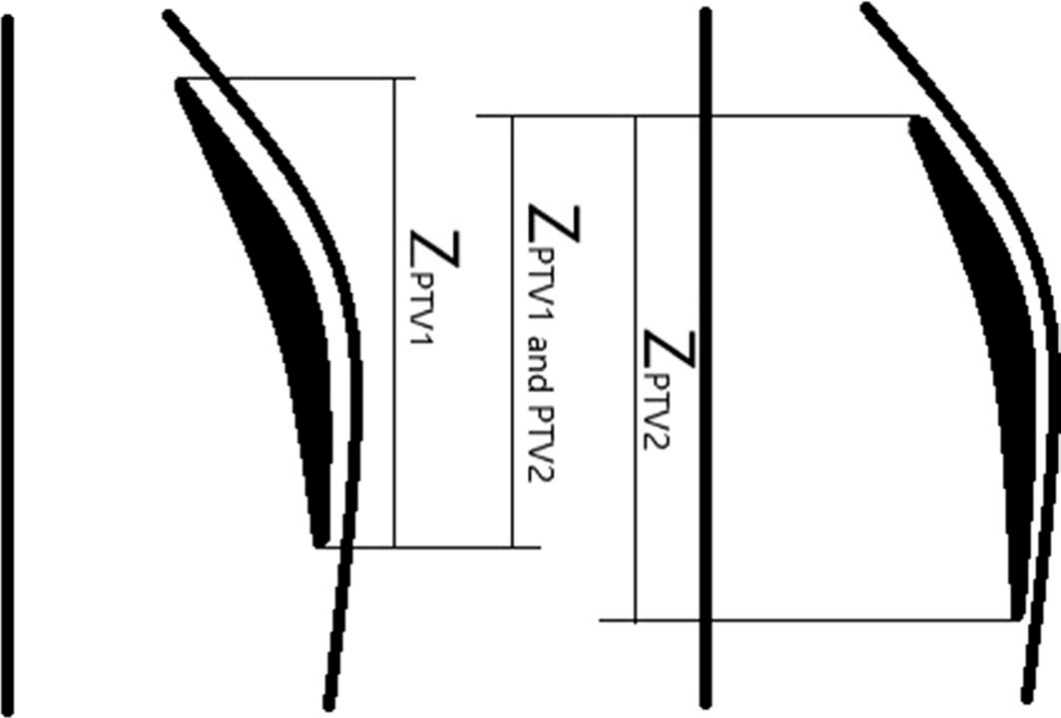
An example of $P_{\text{ovz}}$. Here are two coronal diagrams, and the two images had been aligned through rigid registration

In coplanar treatment, the jaws in the head–foot direction can block most of the radiation, so the dose distribution gradient is usually greater in the head–foot direction than in the other directions. Therefore, the relative positions between PTVs in the new image and atlas image in the head–foot direction would have a substantial impact on the dose prediction. A similar concept was also used in the DVH prediction [13–15]. Two features were used to describe the PTV relative positions in the head–foot direction: (5) the length difference between two images and (6) the *z*-axis (head–foot direction) overlap metric. The *z*-axis overlap metric was introduced as follows:

3 $${\text{P}}_{\text{ovz}} = \frac{{2 \times Z_{{{\text{PTV}}1\; {\text{and}}\; {\text{PTV}}2}} }}{{Z_{{{\text{PTV}}1}} + Z_{{{\text{PTV}}2}} }},$$

where $Z_{{{\text{PTV1}}\; {\text{and }}\;{\text{PTV2}}}}$ is the *z*-axis overlap length of two PTVs, and $Z_{\text{PTV1}}$ and $Z_{\text{PTV2}}$ are the z-axis lengths of two PTVs. Similarly to feature (2), feature (6) was calculated following rigid alignment between two images, and PTV2 in Eq. (3) was affine transformed from the original image during the rigid alignment. The overlap in the z-axis is illustrated in Fig. 2.

Equations (7) to (11): 3DMIs were used to quantify the spatial characteristics in the shape of PTVs. 3DMIs are mathematical shape descriptors that are designed to be invariant to scaling, translation, and rotation [35, 36]. 3DMIs are combinations of terms describing the variance, skewness, and kurtosis of a distribution, and are often used in visual pattern recognition. For a 3D distribution, in this study a PTV mask image, the moments of order $n = p + q + r$ are given by

4 $$m_{pqr} = \mathop \int \limits_{ - \infty }^{ + \infty } \mathop \int \limits_{ - \infty }^{ + \infty } \mathop \int \limits_{ - \infty }^{ + \infty } x^{p} y^{q} z^{r} f\left( {x,y,z} \right)dxdydz,$$

where $\left( {x,y,z} \right)$ are the spatial coordinates of each voxel. $f\left( {x,y,z} \right) = 1$ when $\left( {x,y,z} \right)$ inside the target area and $f\left( {x,y,z} \right) = 0$ when $\left( {x,y,z} \right)$ outside the target area. The first-order moments can be used to find the centroid coordinates of the object in each direction as follows:

5 $$\bar{x} = \frac{{m_{100} }}{{m_{000} }}, \quad \bar{y} = \frac{{m_{010} }}{{m_{000} }}, \quad \bar{z} = \frac{{m_{001} }}{{m_{000} }}.$$

To obtain invariance to position in the image, central moments are used and are defined as follows:

6 $$\mu_{pqr} = \mathop \int \limits_{ - \infty }^{ + \infty } \mathop \int \limits_{ - \infty }^{ + \infty } \mathop \int \limits_{ - \infty }^{ + \infty } \left( {x - \bar{x}} \right)^{p} \left( {y - \bar{y}} \right)^{q} \left( {z - \bar{z}} \right)^{r} \cdot f\left( {x,y,z} \right)dxdydz.$$

To eliminate the influence of PTV volume, the moments are commonly normalized by

7 $$\eta_{pqr} = \frac{{\mu_{pqr} }}{{\mu_{000}^{{\frac{p + q + r}{3} + 1}} }}.$$

Finally, the normalized central moments need to be combined specifically to obtain invariance to rotation. Here, the definitions of Sadjadi and Hall [37] for three second-order moments and of Ng et al. [38] for third- and fourth-order moments invariant to rotation were used as follows:

8 $$\begin{aligned} & J_{1} = \eta_{200} + \eta_{020} + \eta_{002} , \\ & J_{2} = \eta_{200} \eta_{020} + \eta_{200} \eta_{002} + \eta_{020} \eta_{002} - \eta_{101}^{2} - \eta_{110}^{2} - \eta_{011}^{2} , \\ & J_{3} = \eta_{200} \eta_{020} \eta_{002} - \eta_{002} \eta_{110}^{2} - \eta_{020} \eta_{101}^{2} - \eta_{200} \eta_{011}^{2} + 2\eta_{110} \eta_{101} \eta_{011} , \\ & B_{3} = \eta_{300}^{2} + \eta_{030}^{2} + \eta_{003}^{2} + 3\eta_{210}^{2} + 3\eta_{201}^{2} + 3\eta_{120}^{2} + 6\eta_{111}^{2} + 3\eta_{102}^{2} + 3\eta_{021}^{2} + 3\eta_{012}^{2} , \\ & B_{4} = \eta_{400}^{2} + \eta_{040}^{2} + \eta_{004}^{2} + 4\eta_{310}^{2} + 4\eta_{301}^{2} + 6\eta_{220}^{2} + 12\eta_{211}^{2} + 6\eta_{202}^{2} + 4\eta_{130}^{2} + 12\eta_{121}^{2} + 12\eta_{112}^{2} + 4\eta_{103}^{2} + 4\eta_{031}^{2} + 6\eta_{022}^{2} + 4\eta_{013}^{2} . \\ \end{aligned}$$

The differences of five 3DMIs between two PTVs were considered as features for describing the geometric similarities of target areas.

With the exception of features (2) and (6), all of the values of the features decreased when the two patients were more similar. To maintain the consistency of the numerical change direction, all values of features (2) and (6) were replaced with the reciprocals of the original values.

#### Feature selection

To maintain simplicity in the atlas-selection method, features without significant correlations with the prediction results could be excluded to select the atlas image. The Spearman’s rank correlation was used to evaluate the correlations among the 11 features and dose prediction result measures introduced in Validation study. The statistical analysis was performed within the 20 atlas cases. The correlation significances were assessed by the p value. The correlations with p < 0.05 were considered as significant. Furthermore, factor and correlation analyses were applied to characterize the correlated features with less factors. All the correlated features were tested with Bartlett’s sphericity test and the Kaiser–Meyer–Olkin (KMO index). If the KMO measure > 0.5 and the Bartlett’s sphericity significance < 0.05, factor analysis would be used to decrease the feature number. Factor analysis is a statistical method that is used to describe variability among observed, correlated variables in terms of a potentially lower number of unobserved variables known as factors. The factor analysis used principal components to extract the maximum variance from the features. To minimize the number of features with high loadings on any given factor, a varimax rotation was utilized, whereby the factors with eigenvalues less than 1 were excluded. A comprehensive score as a linear combination of the remaining factors was calculated to quantify the patient similarities. The comprehensive score was defined as

9 $$F = \frac{{\sum\nolimits_{i} {\lambda_{i} F} }}{{\sum\nolimits_{i} {\lambda_{i} } }}$$

where $i$ is the number of remaining factors and $\lambda_{i}$ represents the eigenvalues of $F_{i}$. Finally, the quantitative index was used as a gauge for the atlas image selection. All of the mathematical manipulations were performed with SPSS v19 (IBM, Armonk, NY, USA).

### Atlas-selection models

For the cases that were already planned, each voxel in the image had a corresponding dose value. These images were referred to as atlas images, while the image to be predicted was referred to as the new image. The atlas image coordinates were mapped by deformable registration onto new images, thereby providing the dose distribution of the latter. The dose distribution matrix of the atlas image was denoted by $$\overset{\lower0.5em\hbox{$\smash{\scriptscriptstyle\rightharpoonup}$}}{\text{D}}_{\text{atlas}}$$. The transform function following image registration was $$f_{P} (f_{D} (f_{R} ()))$$, while the predicted dose distribution matrix of the new image could be calculated by $$\overset{\lower0.5em\hbox{$\smash{\scriptscriptstyle\rightharpoonup}$}}{\text{D}}_{\text{predict}} = f_{P} \left( {f_{D} \left( {f_{R} \left( {\overset{\lower0.5em\hbox{$\smash{\scriptscriptstyle\rightharpoonup}$}}{\text{D}}_{\text{atlas}} } \right)} \right)} \right)$$. During the image registration procedure, the new image acted as the fixed image, while the atlas image acted as the floating image. Two strategies were introduced to generate the predicted dose distribution, as follows:

#### Most similar image from database (SIM)

**Fig. 3:**
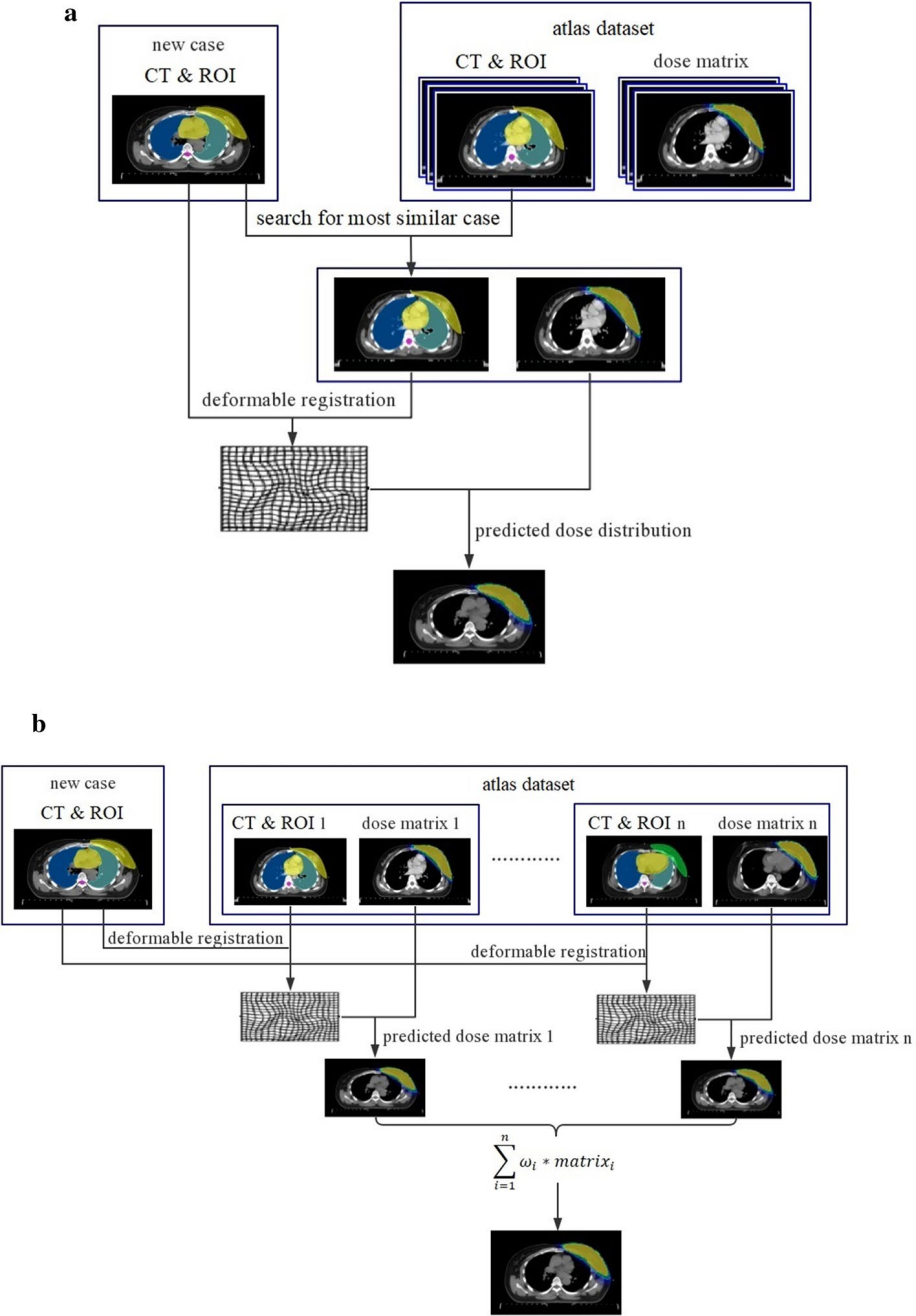
Flowchart of major steps in dose distribution prediction process: **a** SIM and **b** WEI

The new image was rigidly registered to all the images in the atlas database, and the patient similarity metric factors between the new image and each database image were calculated individually. The image with the maximum factors was considered as the most similar atlas image. Thereafter, the most similar atlas image was used for dose prediction. Figure 3a presents a flowchart of the major steps in the process.

#### All images from database with weighted dose distribution (WEI)

The new image was deformably registered to the database images, and each registration produced a dose distribution. The set of all registrations was combined into a probabilistic dose that assigned a probability distribution of the dose value to each voxel in the new image. This probabilistic atlas was reduced to a deterministic atlas by assigning the dose that received the weighted average among all database images to each voxel. Figure 3b presents a flowchart of the major steps in the process. The dose value in voxel *i* was defined as

10 $$d_{i} = \mathop \sum \limits_{j = 1}^{n} \omega_{j} d_{j} ,$$

where *n* is the total number of images in the database, $d_{j}$ is the dose transformed from floating image *j*, and $\omega_{j}$ is the weight factor. Two types of definition methods for $\omega_{j}$ were used in this study. In the first, $\omega_{j}$ was defined as $1/{\text{n}}$, which means that all n atlas images contributed equally to the new dose. In the second, $\omega_{j}$ was calculated from the comprehensive score $F_{j}$, described in Sect. “Feature selection”. First, $F_{j}$ was scaled to the range of (0, 1) using the following function:

11 $$F_{j}^{'} = \frac{{F_{ \text{max} } - F_{j} }}{{F_{ \text{max} } - F_{ \text{min} } }},$$

where $F_{ \text{max} }$ and $F_{ \text{min} }$ are the maximum and minimum of $F_{j}$ between all atlas images and new images. Because $F_{j}$ decreased as the similarity between two images increased, $F_{ \text{min} }$ was the comprehensive score for evaluating the similarity between the new image and most similar atlas image. This transformation caused $F_{j}^{'}$ to be in the range of (0, 1). For a given database of atlas images, $F_{j}^{'} = 1$ when $j$ was the most similar atlas image to the new image, and $F_{j}^{'} = 0$ when $j$ was the least similar atlas image. Thereafter, $\omega_{j}$ was defined as

12 $$\omega_{j} = \frac{{F_{j}^{'} }}{{\mathop \sum \nolimits_{k = 1}^{n} \left( {F_{k}^{'} } \right)}},$$

where n is the total number of atlas images. This definition caused the atlas image that was more similar to the new image to contribute a greater weight when the final result was calculated.

### Validation study

The predicted dose distribution was compared to the clinical plan dose for every registration. As one dose measure predicted the quality, the mean absolute difference (MAD) was measured for the PTV, heart, whole lung, ipsilateral lung, and spinal cord. The MAD for a given ROI was defined as

13 $${\text{MAD}}\left( {\text{ROI}} \right) = \frac{1}{n}\mathop \sum \limits_{i = 1}^{n} \left( {\left| {Dp_{i} - D_{i} } \right|} \right),$$

where $n$ is the number of voxels in the ROI, and $Dp_{i}$ and $D_{i}$ are the predicted and clinical plan doses for voxel $i$, respectively.

As a second measure of segmentation quality, the DSC was computed for 80% and 100% of the prescription dose area. For a given dose, the DSC was defined as

14 $${\text{DSC}}\left( {\text{dose}} \right) = \frac{{2\left( {V_{p} \cap V_{m} } \right)}}{{V_{p} + V_{m} }},$$

where $V_{p}$ is the area of the predicted dose that is larger than the given dose, and $V_{m}$ is the area of the clinical plan dose that is larger than the given dose. For perfect prediction, the DSC had a value of 1. A lesser overlap resulted in smaller DSC values.

The 3D gamma analysis metric was also used to measure the dose distribution similarity quantitatively [39, 40]. The gamma metric between a predicted dose-to-voxel $d_{p}$ and a clinical plan dose is defined as $d_{m}$ in point $r_{m}$ defined as

15 $$\gamma \left( {r_{m} } \right) = { \text{min} }\left\{ {\sqrt {\frac{{\left| {r_{p} - r_{m} } \right|^{2} }}{{\Delta r_{M}^{2} }} + \frac{{\left| {d_{p} - d_{m} } \right|^{2} }}{{\Delta d_{M}^{2} }}} } \right\}\forall \left\{ {r_{p} } \right\}.$$

where $r_{p}$ is a search over a neighborhood of voxels in the predicted dose space $d_{p}$, $\Delta r_{M}$ the spatial distance threshold criterion, and $\Delta d_{M}$ the dose difference threshold criterion. The gamma factor between two distributions is the percentage of voxels with $\gamma \left( {r_{m} } \right) \le 1$, which is the percentage of voxels with dose difference less than $\Delta d_{M}$ to at least one voxel in a spatial no larger than $\Delta r_{M}$ in the predicted dose image. To concentrate on the most crucial area of dose clinically including PTV coverage and dose falloff at the periphery of PTV, the gamma at 80% of prescription dose area was assessed at tolerance levels of $\Delta r_{M} = 3\;{\text{mm}}$, $d_{p} = 3\%$ (33γ) and $\Delta r_{M} = 5\;{\text{mm}}$, $d_{p} = 5\%$ (55γ), respectively.

To determine whether the evaluation parameters were statistically different among the different atlas generation methods, a Friedman test was performed, which is the nonparametric alternative to one-way analysis of variance with repeated measures. If the hypothesis of equal groups was rejected (p < 0.05), post hoc analysis was performed to compare the differences among groups using a nonparametric exact Wilcoxon signed rank test. A significance level of p = 0.05 was used for this test. Statistical analyses were conducted using SPSS v19 (IBM, Armonk, NY, USA).

## Data Availability

The datasets used and/or analyzed during the current study are available from the corresponding author on reasonable request.

## Abbreviations

IMRT: Intensity-modulated radiotherapy
VMAT: Volumetric-modulated arc therapy
OAR: Organ of interest
TPS: Treatment planning system
DVH: Dose–volume histogram
3D: Three-dimensional
CT: Computed tomography
ROIs: Regions of interest
DIR: Deformable image registration
3DMI: 3D moment invariants
CTV: Clinical target volume
PTV: Plan target volume
MSD: Mean squared deviation
DSC: Dice similarity coefficient
OVH: Overlap volume histogram
KMO: Kaiser–Meyer–Olkin
SIM: Most similar image from database
WEI: All images from database with weighted dose distribution
MAD: Mean absolute difference
WEI_A: Average weight combination
WEI_F: Metric weight combination
